# Urologische Prostatakrebsvorsorge im Rahmen der Movember-Gesundheitsinitiative 2019 am Universitätsklinikum Frankfurt

**DOI:** 10.1007/s00120-020-01265-y

**Published:** 2020-07-02

**Authors:** M. Wenzel, C. Humke, S. Wicker, J. Mani, T. Engl, G. Hintereder, T. J. Vogl, P. Wild, J. Köllermann, C. Rödel, S. Asgharie, L. Theissen, M. Welte, L. A. Kluth, P. Mandel, F. K. H. Chun, F. Preisser, A. Becker

**Affiliations:** 1 Klinik für Urologie, Universitätsklinikum Frankfurt, Theodor-Stern-Kai 7, 60590 Frankfurt, Deutschland; 2 Betriebsärztlicher Dienst, Universitätsklinikum Frankfurt, Frankfurt, Deutschland; 3 Urogate Praxis, Frankfurt, Deutschland; 4 Zentrallabor, Zentrum der Inneren Medizin, Universitätsklinikum Frankfurt, Frankfurt, Deutschland; 5 Zentrum für diagnostische und interventionelle Radiologie, Universitätsklinikum Frankfurt, Frankfurt, Deutschland; 6 Dr. Senkenbergisches Institut für Pathologie, Universitätsklinikum Frankfurt, Frankfurt, Deutschland; 7 Klinik für Strahlentherapie und Onkologie, Universitätsklinikum Frankfurt, Frankfurt, Deutschland

**Keywords:** Prostataspezifisches Antigen, PSA-Screening, Vorsorgeuntersuchung, Prävention, Öffentlichkeit, Prostata-specific antigen, PSA screening, Preventive medicine, Cancer check up, Awareness campaign

## Abstract

**Hintergrund:**

Männer in Deutschland sterben früher als Frauen und nehmen weniger häufig Krebsvorsorgeuntersuchungen wahr.

**Fragestellung:**

Ziel war die prospektive Evaluation einer „Movember-Gesundheitsinitiative“ am Universitätsklinikum Frankfurt (UKF) im November 2019.

**Methoden:**

Im Rahmen der „Movember-Gesundheitsinitiative“ wurde allen männlichen Mitarbeitern des UKF ab dem 45. Lebensjahr und bei erstgradiger familiärer Vorbelastung eines Prostatakarzinoms ab dem 40. Lebensjahr im November 2019 gemäß S3-Leitlinien der Deutschen Gesellschaft für Urologie (DGU) eine Prostatakarzinom-Vorsorgeuntersuchung angeboten.

**Ergebnisse:**

Insgesamt nahmen 14,4 % der Mitarbeiter teil. Eine familiäre Vorbelastung gaben insgesamt 14,0 % Teilnehmer an. Das mediane Alter betrug 54 Jahre. Der mediane PSA(prostataspezifisches Antigen)-Wert lag bei 0,9 ng/ml, der mediane PSA-Quotient bei 30 %. Bei 5 % (*n* = 6) zeigte sich ein suspekter Tastbefund in der DRU (digital-rektale Untersuchung). Nach Altersstratifizierung (≤ 50 vs. > 50 Lebensjahre) zeigten sich signifikante Unterschiede im medianen PSA-Wert (0,7 ng/ml vs. 1,0 ng/ml, *p* < 0,01) und der bereits zuvor durchgeführten urologischen Vorsorge (12,1 vs. 42,0 %, *p* < 0,01). Vier Teilnehmer (3,3 %) zeigten erhöhte Gesamt-PSA-Werte. Bei 32,2 % der Teilnehmer zeigte sich mindestens ein kontrollbedürftiger Befund. Insgesamt wurden 6 Prostatabiopsien durchgeführt. Hierbei zeigte sich in einem Fall ein intermediate-risk Prostatakarzinom (Gleason 3 + 4, pT3a, pPn1, pNx, R0).

**Schlussfolgerungung:**

Im Rahmen der UKF-Movember-Gesundheitsinitiative 2019 konnten durch ein Vorsorgeangebot 121 Männer für eine Prostatakrebs-Vorsorge inklusive PSA-Testung gewonnen werden. Auffällige/kontrollbedürftige Befunde zeigten sich bei 32,2 %. Bei einem Mitarbeiter wurde ein therapiebedürftiges Prostatakarzinom entdeckt und therapiert.

## Abstract (en)

**Background:**

Men die earlier than women in Germany. Men also have impaired access to cancer screening compared to women.

**Objectives:**

Our Movember campaign 2019 at University Hospital Frankfurt (UKF) aimed at improving health care awareness in the context of prostate cancer checkup.

**Materials and methods:**

In November 2019, every male employee of the UKF with a minimum age of 45 yrs (or 40 yrs with a first degree relative with prostate cancer) was offered a free prostate cancer checkup. This checkup contained digital rectal examination (DRE), transrectal ultrasound and PSA (prostata-specific antigen) testing.

**Results:**

Overall, 121/840 employees (14.4%) participated in the Movember campaign. A first degree relative with prostate cancer was reported in overall by 14% of the participants (*n* = 17). At least one prior prostate cancer check up had 33%. A total of 2.5% (*n* = 3) had one prior negative prostate biopsy. Median age was 54 yrs (interquartile range 50–58). Median PSA level was 0.9 ng/ml and median free-PSA 0.3 ng/ml. A suspicious DRE was found in 5% (*n* = 6). After stratification according to age (≤ 50 yrs vs. > 50 yrs), participants over 50 yrs had a significantly higher PSA level (1.0 ng/ml vs. 0.7 ng/ml, *p* < 0.01) and had more frequently at least one prior prostate cancer checkup in the past (42.0 vs. 12.1%, *p* < 0.01). All suspicious DREs were in the cohort > 50 yrs. Overall, 32.2% (*n* = 39) had at least a suspicious checkup. A total of 3.3% (*n* = 4) had suspicious PSA levels. 17.4% (*n* = 21) of the participants had a suspicious PSA ratio (< 20%) only. During follow-up, 6 prostate biopsies were performed, with the detection of one case of intermediate-risk prostate cancer (Gleason 3 + 4, pT3a, pPn1, pNx, R0).

**Conclusion:**

Overall, 121 employees participated in our Movember Prostate cancer checkup campaign with measurement of the PSA level. Suspicious results were recorded in 32.2%. One employee was diagnosed and successfully treated with an intermediate-risk prostate cancer.

## Einleitung

Männer sterben früher als Frauen. Die durchschnittliche Lebenserwartung von Männern und Frauen in Deutschland beträgt aktuell etwa 78 gegenüber 83 Jahre [24]. Männer nehmen im Vergleich zu Frauen weniger häufig Krebsvorsorgeuntersuchungen wahr [6]. Das Prostatakarzinom ist die häufigste Krebserkrankung und eine der häufigsten Todesursachen bei Männern in Deutschland und Europa [8, 9, 24], welche zudem eine um 6 % höhere krebsspezifische Sterblichkeit haben [18].

Die Movember-Foundation ist die weltweit größte „Crowd-founding-Organisation“, mit dem Ziel, die Aufmerksamkeit und Akzeptanz für das Thema Männergesundheit zu erhöhen [17]. Hierfür wird jährlich der November zum Aktionsmonat „Movember“ (Kunstwort aus „Moustache“ und November) ausgerufen und Männer aufgefordert, als Zeichen der Solidarität einen Oberlippenbart zu tragen und für dieses Thema zu spenden. Die Movember-Foundation fördert mit diesen Mitteln weltweit renommierte Forschungsprojekte [13].

Ziel dieser Arbeit war es, im Rahmen der Movember-Gesundheitsinitiative 2019 eine klinikuminterne Prostatakrebsvorsorge am Universitätsklinikum Frankfurt zu etablieren und prospektiv zu evaluieren.

## Material und Methoden

Nach positivem Ethikvotum (Nr. 19-400) wurde im Rahmen der Movember-Gesundheitsinitiative 2019 am Universitätsklinikum Frankfurt im Zeitraum 01.11.–30.11.2019 allen männlichen Mitarbeitern ab dem 45. Lebensjahr und bei erstgradiger familiärer Vorbelastung eines Prostatakarzinoms ab dem 40. Lebensjahr (gemäß der deutschen S3-Leitlinie; [11]) eine kostenlose urologische Prostatakrebsvorsorgeuntersuchung angeboten. Die Initiative wurde mittels Aushängen und E‑Mail-Ankündigungen durch den Klinikumsvorstand befürwortet, angekündigt und beworben. Die Durchführung erfolgte unter Federführung der Klinik für Urologie und wurde durch den betriebsärztlichen Dienst, das Zentrallabor, das Institut für Pathologie, die Kliniken für Radiologie, Strahlentherapie sowie ein Kooperationsnetzwerk niedergelassener Urologen und durch die Movember-Foundation unterstützt. Die Vorsorgeuntersuchung beinhaltete eine Anamnese mit Erhebung bereits durchgeführter Vorsorgen, Vorerkrankungen, vormaligen PSA-Werten, Biopsien und bildgebenden Untersuchungen der Prostata. Vor der klinischen Untersuchung (KU) wurde der absolute PSA-Wert (tPSA) und das freie PSA (fPSA) sowie der PSA-Quotient (prozentuales Verhältnis aus fPSA/tPSA = %fPSA-Quotient) bestimmt. Die KU beinhaltete die digital-rektale Untersuchung (DRU) sowie einen transrektalen Ultraschall (TRUS). Die Vorsorge wurde durch urologische Assistenz‑/Fachärzte sowie von zwei niedergelassenen Urologen durchgeführt.

### Datenschutz – Pseudonymisierung

Nach schriftlicher Aufklärung und Einwilligung zur Teilnahme wurden alle Teilnehmer per Zufallsverfahren numerisch pseudonymisiert. Alle erhobenen Befunde wurden auf entsprechenden vorgefertigten Anamnese- oder Untersuchungsbögen schriftlich unter dem jeweiligen, individuellen Pseudonymisierungskode (PC) erhoben. Eine Aufnahme von Patientendaten oder Untersuchungsbefunden in das klinikinterne System wurde somit vermieden. Als Datentreuhänderin diente die Leiterin (S.W.) des betriebsärztlichen Diensts. Nur ihr war eine Entpseudonymisierung des PC möglich.

### Auswertung, Entpseudonymisierung und Befundübermittlung

Die Auswertung der gewonnenen Daten erfolgte anhand des pseudonymisierten Erhebungsbogens. In Zusammenschau aller Befunde (Alter, familiärer Vorbelastung, Vorbefunde der Anamnese, klinischer Untersuchung, Sonographie und PSA-Werte) wurde für jede Teilnehmernummer ein individualisierter Befundbericht und Empfehlung über das weitere Prozedere erarbeitet. Diese Movember-2019-Empfehlungen wurden an die Datentreuhänderin des betriebsärztlichen Dienstes übermittelt, dann erfolgte die Entpseudonymisierung des PC und die urologischen Empfehlungen wurden postalisch an die Teilnehmer verschickt.

In der Folge konnten die Teilnehmer auf Wunsch einen Termin in unserer Prostatakarzinomspezialsprechstunde zur Kontrolle oder Befundbesprechung, nach vorheriger Einwilligung zur Entpseudonymisierung und Movember-Befundsichtung, vereinbaren. Die dabei erhobenen Befunde wurden als Follow-up-Daten ausgewertet.

### Statistik

Die deskriptive Statistik beinhaltet Proportionen, Prozentzahlen, Mediane und den Interquartilsabstand (IQR). Eine Auswertung von Signifikanzen hinsichtlich des Alters (≤50 vs. >50 Jahre, 50 Jahre als 25 %-Quartilsgrenze gewählt) wurde zum α = 5 % Niveau mittels χ^2^-Test für kategorische Variablen bzw. mittels Mann-Whitney-U-Test für Proportionen durchgeführt. LOESS-Plots und lineare Regressionsmodelle wurden verwendet, um den Zusammenhang zwischen PSA-Werten und dem Lebensalter zu überprüfen. Hierfür wurde die Statistiksoftware R statistics (Version 3.4.4, RStudio, Boston, USA) verwendet.

## Ergebnisse

Insgesamt arbeiten am Universitätsklinikum Frankfurt 840 männliche Mitarbeiter ≥45 Jahren, wovon 121 Mitarbeiter (14,4 %) an der Movember-Gesundheitsinitiative teilnahmen. Eine familiäre Vorbelastung für ein Prostatakarzinom gaben 17 von 121 Teilnehmer (14,0 %) an, wovon 6 Teilnehmer <45 Jahre alt waren (5 % der Gesamtteilnehmer). Eine urologische Vorsorgeuntersuchung vor der Teilnahme an der Movember-Gesundheitsinitiative 2019 hatten bereits 41 (33,9 %) Teilnehmer. Bei keinem wurde zuvor ein multiparametrisches MRT (mpMRT) der Prostata durchgeführt. Eine jeweils einmalige vorherige negative Prostatabiopsie hatten 3 (2,5 %) Teilnehmer.

Das mediane Teilnehmeralter betrug 54 Jahre (IQR 50–58 Jahre; Tab. 1). Der mediane tPSA lag bei 0,9 ng/ml (IQR 0,6–1,7 ng/ml). Der mediane %fPSA-Quotient lag im Gesamtkollektiv bei 30 % (IQR 20–40 %), bei einem medianen fPSA von 0,3 ng/ml (IQR 0,2–0,4 ng/ml). In der KU zeigte sich bei 6 (5 %) Teilnehmern ein suspekter Tastbefund in der DRU. Das mediane Prostatavolumen betrug im TRUS 26 cm^3^ (IQR 21–35 cm^3^). Unscharfe Prostatarandkonturen wurden in einem Fall (0,8 %) im TRUS beschrieben, auffällige Samenbläschen, z. B. im Sinne einer Vergrößerung, bei 6 (5 %) Teilnehmern (Tab. 2).

**Table 1:** 

Variable	Gesamt	Alter >50 Jahre	Alter ≤50 Jahre	*p*-Wert
Probanden (*n* [%])	121	88 (72,7)	33 (27,3)	–
Alter (Jahre), Median (IQR)	54 (50–58)	56 (54–60)	46 (45–48)	<0,001
PSA gesamt (ng/ml) Median (IQR)	0,9 (0,6–1,7)	1,0 (0,7–2,1)	0,7 (0,5–0,9)	0,001
Freies PSA (ng/ml) Median (IQR)	0,3 (0,2–0,4)	0,3 (0,2–0,4)	0,2 (0,2–0,4)	0,1
PSA-Quotient (%) Median (IQR)	30 (20–40)	30 (20–30)	30 (30–40)	0,002
TRUS P‑Volumen (cm^3^), Median (IQR)	26 (21–35)	30 (22–36)	22 (20–26)	<0,001
DRU Suspekt^a^ (*n* [%])	6 (5,0)	6 (6,8)	0 (0)	0,3
Positive Familienanamnese PCA (*n* [%])	18 (14,9)	9 (10,2)	9 (27,3)	0,1
Vorherige Vorsorge (*n* [%])	41 (33,9)	37 (42)	4 (12,1)	0,007
Vorherige Biopsie (*n* [%])	3 (2,5)	3 (3,4)	0 (0)	0,5

*PSA* prostataspezifisches Antigen, *TRUS* transrektaler Ultraschall, *P‑Volumen* Prostatavolumen, *DRU* digital-rektale Untersuchung, *PCA* Prostatakarzinom

^a^Missing Data: *n* = 3

**Table 2:** 

Variable	Gesamt	Alter ≤50 Jahre	Alter >50 Jahre	*p*-Wert
Unauffällige Vorsorge, *n* (%)	82 (67,8)	29 (87,9)	53 (60,2)	0,1
Kurzfristige Kontrollempfehlung wegen absoluter PSA-Wert Erhöhung oder auffälliger DRU (*n* [%])^a^	8 (6,6)	0 (0)	8 (9,1)	–
Zeitnahe Kontrollempfehlung wegen auffälligem PSA-Quotienten (<20 %), (*n* [%])	21 (17,4)	3 (9,1)	18 (20,5)	–
Kontrollempfehlung wegen TRUS-Befund (*n* [%])^a^	8 (6,6)	1 (3,0)	7 (7,9)	–
Kontrollempfehlung wegen PSA-Dynamik zu Vorbefunden (*n* [%])^a^	2 (1,7)	0 (0)	2 (2,3)	–

*PSA* prostataspezifisches Antigen, *DRU* digital-rektale Untersuchung, *TRUS* transrektaler Ultraschall

^a^Zum Teil in Kombination mit zusätzlich auffälligem PSA-Quotienten <20 %

**Figure 1:**
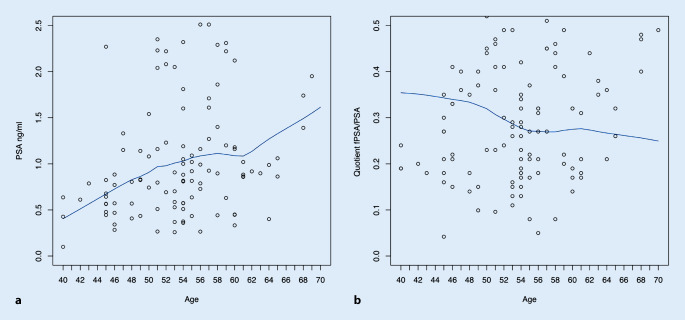

Es erfolgte eine Stratifizierung der Teilnehmer bezüglich des Alters in ≤50 vs. >50 Jahre. Hierbei zeigten sich signifikante Unterschiede im medianen tPSA-Wert (0,7 vs. 1,0 ng/ml, *p* = 0,001), dem %fPSA-Quotienten (30 % [IQR 20–30 %] vs. 30 % [IQR 30–40 %], *p* = 0,002) und der Häufigkeit bereits ambulanter durchgeführter urologischer Vorsorge (12,1 vs. 42,0 %, *p* = 0,007). Ebenso zeigte sich in den Regressionsanalysen eine signifikante Korrelation zwischen dem Lebensalter und einem tPSA-Anstieg (*p* < 0,001) sowie einer Abnahme des %fPSA-Quotienten (*p* = 0,02, Abb. 1). Auffällige Tastbefunde und bereits im Vorfeld durchgeführten Biopsien waren ausschließlich dem Kollektiv von >50 Jahren vorbehalten.

### Kontrollempfehlungen

Entsprechend der erhobenen Befunde erhielt jeder Teilnehmer der UKF-Movember-Gesundheitsinitiative 2019 eine individualisierte Empfehlung. Bei 82 (67,8 %) zeigten sich unauffällige Befunde, sodass lediglich eine Empfehlung zur nächsten klinischen Vorsorgeuntersuchung nach einem Jahr ausgesprochen wurde. Vier (3,3 %) Teilnehmer zeigten erhöhte tPSA-Werte (6,0, 6,6, 5,7 und 4,2 ng/ml), sodass die Empfehlung zur zeitnahen Kontrollbestimmung mit ggf. Durchführung einer Prostatabiopsie lautete. Bei rein auffälligen %fPSA-Quotienten (<20 % und tPSA <2,5 ng/ml [16, 28], 17,4 %, *n* = 21) wurde eine Kontrollbestimmung und Reevaluation der klinischen und laborchemischen Untersuchung innerhalb der nächsten 3 Monate empfohlen, ebenso wie bei Teilnehmern mit auffälligen Tast- (3,3 %, *n* = 4) oder TRUS-Befunden (6,6 %, *n* = 8), sowie starker PSA-Dynamik (>1 ng/ml/Jahr) zu Vorwerten (1,7 %, *n* = 2), teilweise in Kombination mit %fPSA-Quotienten <20 %.

### Kontrolluntersuchungen

Insgesamt 15 (12,4 %) Teilnehmer stellten sich innerhalb der nächsten 3 Monate auf eigenen Wunsch in unserer Prostatakarzinomspezialsprechstunde zur persönlichen Befundbesprechung und klinischen/laborchemischen Kontrolle vor. Hierbei zeigte sich bei 6 Patienten eine unauffällige klinische und/oder laborchemische Nachkontrolle. Insgesamt wurde in der Nachkontrolle bei 9 Patienten (7,4 %) mit auffälligen tPSA-Wert oder -%fPSA ein mpMRT der Prostata durchgeführt. PI-RADS-1- bis -2- vs. PI-RADS-3- vs. PI-RADS-4-Befunde zeigten sich bei 2 vs. 5 vs. 2 Patienten. Bei keinem Teilnehmer konnte eine PI-RADS-5-Läsion erhoben werden. Bei 6 (5,0 %) Patienten wurde nach eingehender Beratung entsprechend der Wahrscheinlichkeit für das Vorliegen eines signifikantes Prostatakarzinom, entsprechend der PRECISION-Studie [12], eine fusionierte Prostatabiopsie inklusive randomisierter Biopsie durchgeführt. Ein Patient entschied sich gegen eine Biopsie. Interessanterweise konnte bei einem Patienten (0,8 %) mit PI-RADS-3-Läsion ein Low-risk-Prostatakarzinom nach d’Amico ([4]; iPSA 5,71 [%fPSA-Quotient: 8 %], DRU: cT1c, Gleason 3+3, Tumornachweis in 3/14 Proben mit maximaler 10 % Tumorinfiltration) nachgewiesen werden. Nach eingehender Therapieoptionenberatung wurde eine roboterassistierte beidseits nervschonende radikale Prostatektomie (mittels Neurosave- und „Full functional length urethral sphincter preservation“-Methode [21, 23]) durchgeführt. In der abschließenden Histologie zeigte sich ein signifikantes Adenokarzinom der Prostata (pT3a, pNx, pPn1, R0, Gleason 3+4) mit einem Tumorvolumen von 15 % (größter Einzelherd 1,6 cm; bilateral multifokal ohne abgrenzbaren Indextumor).

## Diskussion

Männer unterziehen sich weniger häufig Krebsvorsorgeuntersuchungen im Vergleich zu Frauen [6]. Geschlechtsspezifische und sozioökonomische Unterschiede in der Wahrnehmung von Krebsvorsorgeuntersuchungen konnten für mehrere verschiedene maligne Erkrankungen nachgewiesen werden [25] Das Prostatakarzinom nimmt aufgrund seiner hohen Prävalenz und Mortalität [8, 9, 24] sowie der Möglichkeit des PSA-basierten Screenings eine Sonderstellung in der Vorsorge ein.

Allerdings wird der Stellenwert des PSA-Screenings sowie die Frequenz und das Alter bei Beginn der Durchführung im Rahmen der Prostatakrebsvorsorge weiterhin kontrovers diskutiert [11, 15, 16]. Möglichkeiten einer adaptierte Risikokalkulation eines entsprechenden Baseline-tPSA werden vermehrt vorgeschlagen [14]. Ein zusätzliches Hemmnis in Deutschland stellt die vorhandene Kostenübernahme für eine PSA-Bestimmung durch die Krankenkassen dar. Eine solche Kostenübernahme wurde zuletzt u. a. von der Europäischen und Deutschen Gesellschaft für Urologie (EAU, DGU) gefordert [5, 7].

In der vorliegenden Studie unterzogen sich 121 Männer mit einem medianen Alter von 54 Jahren einer Prostatakrebsvorsorgeuntersuchung inklusive opportunistischem PSA-Screening. Der mediane tPSA lag bei 0,9 ng/ml bei einem %fPSA-Quotienten von 30 %.

Die erhobenen PSA-Werte sind im Sinne eines opportunistischen PSA-Screenings zu interpretieren, da hier keine systematische Reihenuntersuchung einer kompletten Bevölkerungsschicht erfolgte. Unsere Arbeit stellt somit inhaltlich eine Querschnittsanalyse im Rahmen einer singulären Vorsorgeinitiative dar, die drei für die Prostatakrebsvorsorge wichtige Aspekte beleuchtet:

Erstens bestätigten sich wie erwartet altersabhängige Werte für tPSA und fPSA. Ein signifikant höherer tPSA und %fPSA-Quotient zeigte sich sowohl stratifiziert bei Männern >50 Jahren im Vergleich zu ≤50 Jahren als auch kontinuierlich betrachtet in der Regressionsanalyse mit einem höheren Lebensalter. Interessanterweise zeigten sich keine signifikanten Unterschiede zwischen verschiedenen Bildungsgraden („mindestens Hochschulreife“ vs. „keine Hochschulreife“; Daten nicht gezeigt). Vergleicht man unsere Ergebnisse mit einer ähnlichen Mitarbeiterpräventionskampagne von Chun et al. (*n* = 313) an der Universität Montreal von 2006, zeigen sich ähnliche Charakteristika (medianes Alter 55 Jahre, tPSA 0,8 ng/ml, %fPSA-Quotient 27 %). Insgesamt konnten bei Chun et al. bei 2,2 % der Teilnehmer ein Prostatakarzinom diagnostiziert werden [3].

Zweitens ist der %fPSA-Quotient als alleiniger Marker wenig hilfreich in der Indikationsstellung für eine weitergehende Abklärung. In unserer Studie wiesen 30 Patienten (24,8 %) einen auffälligen PSA-Quotienten (<20 %) auf. Hiervon hatten *n* = 19 eine tPSA <2,5 ng/ml und *n* = 6 bzw. *n* = 5 einen tPSA zwischen 2,5–4 bzw. 4–10 ng/ml. Zwar ist der %fPSA-Quotient laut EAU-Guideline nur für tPSA-Werte zwischen 4–10 ng/ml als Instrument empfohlen, allerdings zeigten Walz et al., dass auch bei einem tPSA von <2,5 ng/ml der %fPSA-Quotient ein hilfreiches Werkzeug zu Diskriminierung für das Vorliegen eines Prostatakarzinoms darstellt [2, 16, 19, 28]. Aufgrund der jedoch geringen Sensitivität (0,7) und Spezifität (0,5) ist eine Indikationsstellung zur bioptischen Klärung aufgrund eines auffälligen %fPSA-Quotienten kritisch zu sehen [10]. Für die Interpretation des Wertes sind daher aus unserer Sicht weitere Parameter wie Alter, Höhe des tPSA und klinische Befunde notwendig und bei isoliert auffälligem %fPSA-Quotienten sollte lediglich eine Kontrolle des tPSA und des %fPSA erfolgen, welches bei entsprechenden Patienten auch nach bestätigender Kontrollmessungen empfohlen wurde.

Drittens bestand durch die Einbindung der Klinik für Radiologie in unserer Studie für Studienteilnehmer mit auffälligen Befunden die – über die aktuellen Leitlinienempfehlung [11] hinausgehende – Möglichkeit zur Durchführung eines mpMRT. Auffällige Läsionen im mpMRT (5 × PI-RADS 3, 2 × PI-RADS 4) wurden entsprechend der PI-RADS-v2-Klassifikation mit spezieller uroradiologischen Befundung klassifiziert. Nach ausführlicher Beratung der Patienten hinsichtlich der Wahrscheinlichkeit für das Vorliegen eines signifikanten Prostatakarzinoms gemäß der PRECISION-Studie [1, 12] konnte bei 2 Patienten aufgrund eines PI-RADS-1/2-Befunds in der mpMRT-Diagnostik auf eine Biopsie verzichtet werden, während bei 6 Patienten eine fusionsbioptische Abklärung erfolgte. Explizit wurde beim Vorliegen einer PI-RADS-3-Läsion das Für und Wider der Prostatabiopsie erörtert. Insgesamt wurde 1 behandlungsbedürftiges Prostatakarzinom diagnostiziert. Die vorliegende Studie kann aufgrund des Studiendesigns und der beschränkten Teilnehmerzahl keinen Aufschluss über die Sinnhaftigkeit des opportunistischen PSA-Screenings als potenziellen Trigger für eine Prostatabiospie oder den Einsatz des mpMRT der Prostata in der Abklärung suspekter Befunde geben.

Allerdings zeigt unsere Studie viertens, dass durch eine koordinierte, interdisziplinäre Gesundheitsinitiative eine signifikante Anzahl von gesunden, asymptomatischen Männern (14,4 %) zu einer betrieblichen Prostatakrebsvorsorge bewogen werden kann. Dies konnte auch bereits in anderem Kontext für das kolorektale und das Mammakarzinom gezeigt werden [20, 22]. Hierbei konnte durch die vermehrte Nutzung sozialer Medien und etablierten „Awareness-Tagen“ (#Worldcancerday) die Aufmerksamkeit in der breiten Bevölkerung hinsichtlich einer notwendigen Vorsorgeuntersuchung deutlich gesteigert werden [26, 27]. Insgesamt könnten ähnlich durchgeführte Gesundheitsinitiativen problemlos in größeren Betriebsstädten und Unternehmen übertragen und etabliert werden.

Zusammenfassend konnten durch unsere klinikuminterne Movember-Gesundheitsinitiative insgesamt 121 Mitarbeiter zur Durchführung einer Prostatakrebsvorsorgeuntersuchung bewegt werden. Dabei wurden bei fast einem Drittel der Patienten kontrollbedürftige Befunde ermittelt und bei einem Patienten ein Prostatakarzinom diagnostiziert. Flankierende Public-awareness-Kampagnen wie die Movember-Aktion können hilfreich sein, die Akzeptanz der Vorsorgeuntersuchung für Männer zu erhöhen.

Limitationen unserer Movember-Gesundheitsvorsorge stellen v. a. die kleine Kohorte sowie das nur für eine Subkohorte vorliegende Follow-up dar. Teilnehmer der Aktion waren v. a. Männer, welche möglicherweise bereits durch ihr berufliches Umfeld an einem Universitätsklinikum einen vereinfachten Zugang zum Gesundheitssystem und erhöhtes Gesundheitsbewusstsein aufweisen und regelmäßige Vorsorge betreiben. Andererseits kann eine Prostatakrebsvorsorge an der eigenen Klinik mit einer gewissen Schambelastung einhergehen. Hierbei kann es durchaus zu einer Selektion unserer Kollektivs gekommen sein. Um diesen Faktor abzumildern, wurden an mehreren Terminen Vorsorgeuntersuchungen durch externe Urologen angeboten.

## Fazit für die Praxis

Öffentliche Gesundheitsinitiativen wie die Movember-Gesundheitsinitiative können helfen, Barrieren zu Krebsvorsorgeuntersuchungen abzubauen.
